# Neuromuscular development in Patellogastropoda (Mollusca: Gastropoda) and its importance for reconstructing ancestral gastropod bodyplan features

**DOI:** 10.1111/jzs.12112

**Published:** 2015-12-03

**Authors:** Alen Kristof, André Luiz de Oliveira, Konstantin G. Kolbin, Andreas Wanninger

**Affiliations:** ^1^ Department of Integrative ZoologyUniversity of ViennaViennaAustria; ^2^ Laboratory of Cell DifferentiationA.V. Zhirmunsky Institute for Marine BiologyFar East Branch of the Russian Academy of SciencesVladivostokRussian Federation

**Keywords:** Evodevo, immunohistochemistry, Lophotrochozoa, muscle system, nervous system, CLSM

## Abstract

Within Gastropoda, limpets (Patellogastropoda) are considered the most basal branching taxon and its representatives are thus crucial for research into evolutionary questions. Here, we describe the development of the neuromuscular system in *Lottia* cf. *kogamogai*. In trochophore larvae, first serotonin‐like immunoreactivity (lir) appears in the apical organ and in the prototroch nerve ring. The arrangement and number of serotonin‐lir cells in the apical organ (three flask‐shaped, two round cells) are strikingly similar to those in putatively derived gastropods. First, FMRFamide‐lir appears in veliger larvae in the *Anlagen* of the future adult nervous system including the cerebral and pedal ganglia. As in other gastropods, the larvae of this limpet show one main and one accessory retractor as well as a pedal retractor and a prototroch muscle ring. Of these, only the pedal retractor persists until after metamorphosis and is part of the adult shell musculature. We found a hitherto undescribed, paired muscle that inserts at the base of the foot and runs towards the base of the tentacles. An apical organ with flask‐shaped cells, one main and one accessory retractor muscle is commonly found among gastropod larvae and thus might have been part of the last common ancestor.

## Introduction

**Figure 1:**
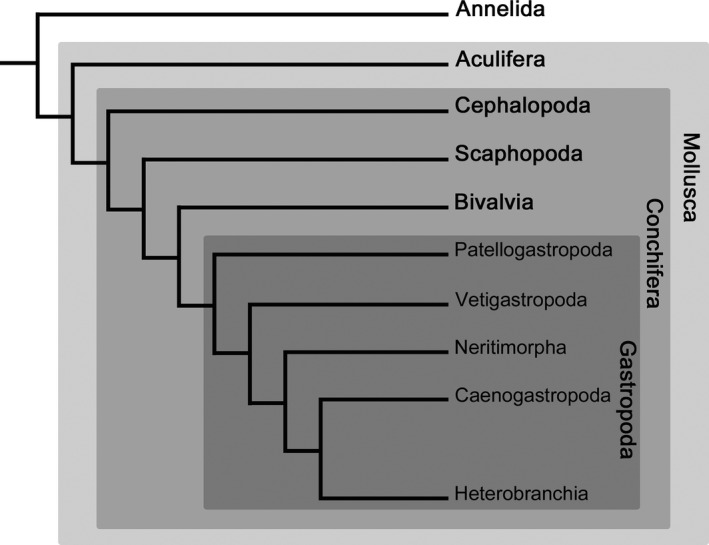
Relationships among major lineages of Mollusca and Gastropoda with Annelida as an outgroup. Topology based on molecular genetic analyses of 308 genes by Kocot et al. (2011). Note that Heterobranchia comprises additionally to Euthyneura (e.g. Nudipleura, Euopisthobranchia, Panpulmonata) also some smaller groups (i.e. Architectonicoidea, Valvatoidea, Rhodopemorpha).

With more than 100 000 extant species Gastropoda is the largest class within Mollusca (Haszprunar et al. 2008). Its representatives are the only molluscs that have adapted to marine, freshwater and terrestrial habitats (Aktipis et al. 2008). The colonization of the different habitats has led to the great morphological diversity seen in extant gastropods. The evolutionary origin of the gastropod bodyplan remains a fundamental question in various disciplines of, for instance, morphology, neurobiology, behavioural, and developmental biology (Haszprunar 1988; Van den Biggelaar and Haszprunar 1996; Katz et al. 2001; Croll and Dickinson 2004; Klussmann‐Kolb et al. 2013). Numerous hypotheses and assumptions about ancestral gastropod bodyplan features have been mostly inspired by adult morphology and some developmental observations. For instance, gastropod relationships were based on studies of single organ systems such as the shell, radula, muscle, and nervous system (Haszprunar 1988; Ponder and Lindberg 1997). Based on these and other morphological and molecular genetic data five major clades, Patellogastropoda, Vetigastropoda, Neritimorpha, Caenogastropoda and Heterobranchia, the latter including euopisthobranchs, nudipleurans and panpulmonates, are recognized within Gastropoda (Fig. 1; Haszprunar 1988; Aktipis et al. 2008; Schrödl 2014). The monophyly of these gastropod lineages are well supported and Patellogastropoda generally appears as the sister taxon to the remaining gastropods (Kocot et al. 2011; Smith et al. 2011; Osca et al. 2014) (but see Grande et al. 2008 for an alternative scenario).

Comparative developmental studies on a number of metazoans have provided important insights into morphogenetic changes that occurred during evolution (Wanninger 2009, 2015a,b). In particular, detailed descriptions of larval neuro‐ and myo‐anatomical characters by immunohistochemistry and F‐actin labelling in conjunction with confocal laser scanning microscopy have facilitated the identification of homologous components of the nervous and muscle system across different invertebrates including gastropods (e.g. Wanninger et al. 1999a,b; Croll and Dickinson 2004; Wollesen et al. 2007, 2008; Evans et al. 2009; Wanninger 2009; Kristof and Klussmann‐Kolb 2010). Accordingly, numerous different representatives from gastropod lineages such as the Caenogastropoda and Heterobranchia are well investigated and data on their neuromuscular development is available (e.g. Bonar and Hadfield 1974; Diefenbach et al. 1998; Page and Parries 2000; Ruthensteiner and Schaefer 2002; Dickinson and Croll 2003; Wollesen et al. 2007, 2008; Kristof and Klussmann‐Kolb 2010; Ruiz‐Jones and Hadfield 2011; Page and Ferguson 2013).

Patellogastropods are almost exclusively marine animals that inhabit predominantly the intertidal and shallow subtidal areas, while some species such as *Eulepetopsis*,* Bathyacmaea,* and *Pectinodonta* also occur in deeper habitats such as on sunken wood or hydrothermal vents (see Lindberg 2008 and references therein). Although patellogastropods have been extensively used in ecological investigations (e.g. Underwood 2000; Lindberg 2008; Range et al. 2008; Underwood et al. 2008) knowledge on their organogenesis including the nervous and muscle system is limited to few investigations (Patelloida: Smith 1935; Wanninger et al. 1999a,b; Damen and Dictus 2002; Lottiidae: Page 2002a). This is particularly surprising, because the basal branching position of Patellogastropoda renders them of prime importance for evolutionary questions (Kocot et al. 2011; Smith et al. 2011; Osca et al. 2014). Herein, we describe the FMRFamide‐like and serotonin‐like immunoreactivity (lir) in the nervous system as well as myogenesis from early larvae to juveniles in the patellogastropod *Lottia* cf. *kogamogai*. In a comparative analysis, possible ancestral features of the gastropod (larval) neuromuscular bodyplan are revealed and discussed.

## Materials and Methods

### Animals

The patellogastropod *Lottia* cf. *kogamogai* was collected from July until August 2011 and 2013 from rocky shores in the Vostok Bay, Peter the Great Bay, Sea of Japan, Russian Federation. These specimens have been initially identified as Lottia kogamogai Sasaki & Okutani, 1994. As molecular genetic analysis shows that they are significantly different in the mitochondrial *cytochrome c oxidase* subunit I (*CO1*) and *16S* rRNA genes and might represent a distinct species (Kristof et al. unpublished) we here refer to this species as *Lottia* cf. *kogamogai*. Adult specimens were collected from intertidal rocks and stones in the vicinity of the marine biological station ‘Vostok’ (approx. 150 km north of Vladivostok, Russian Federation) and were kept in the laboratory in natural seawater at ambient temperature (20–22°C) until gametes were released. Fertilized eggs were transferred to glass bowls and maintained at room temperature (22–24°C) in filtered and UV‐sterilized seawater that was changed once daily until fixation. First cleavage occurred 30 min after fertilization and swimming trochophore larvae were observed after 3–5 h postfertilization (hpf), followed by the veliger (16–20 hpf) and pediveliger stage (24–28 hpf). When larvae increasingly crawled on the bottom (indicating metamorphic competency), mostly on the 3rd day after fertilization, stones and pieces of mussel shells (*Crenomytilus grayanus*) from the collection sites were added to induce metamorphosis. This is a crucial stage, since larvae stagnated in their development if not provided with the right metamorphic cue. The emerging adult shell (teleoconch) became visible in metamorphosing specimens after 5–7 days after fertilization at the base of the larval shell (protoconch). Development was followed until the early postmetamorphic stages (5–7 dpf).

### F‐actin staining and immunolabelling

The seawater was slowly exchanged by adding drops of a 3.5% MgCl_2_ solution until larvae were fully relaxed. In order to avoid a final muscle contraction, a few additional drops of 7% MgCl_2_ solution were added prior to fixation with 4% paraformaldehyde (PFA) in 0.1 M phosphate‐buffered saline (PBS; pH 7.3). The solution was slowly and entirely exchanged with the fixative and kept at room temperature (22–24°C) for 1.5 h followed by three to four washes (15 min each) in 0.1 M PBS (pH 7.3) with 0.1% sodium azide (NaN_3_) added, and stored at 4°C until further processing.

For F‐actin staining, the stored larvae were decalcified in 0.5 M ethylene glycol tetraacetic acid (EGTA) for 1 h and subsequently rinsed multiple times in 0.1M PBS for 6 h. Specimens were then permeabilized and incubated overnight at room temperature (21°C) in a solution containing 4% Triton X‐100 in 0.1 M PBS to which Alexa Flour 488 phalloidin (F‐actin staining; Molecular Probes, Eugene, OR, USA) in a 1 : 40 dilution and 4′, 6‐diamidino‐2‐phenylindole (DAPI) (cell nuclei staining; Molecular Probes) in a 1 : 200 dilution was added. Thereafter, stained specimens were washed three times in 0.1 M PBS at 15 min intervals and mounted in Fluoromount G (Southern Biotech, Birmingham, AL, USA) on glass slides.

After decalcification (see above), permeabilization, and incubation in a blocking solution against non‐specific binding sites (4% Triton X‐100 + 6% normal goat serum (Jackson ImmunoResearch, West Grove, PA, USA) in 0.1 M PBS for 24 h), specimens were incubated in a reaction cocktail with a monoclonal anti‐acetylated‐α‐tubulin antibody (raised in mouse, diluted 1 : 500) (Sigma‐Aldrich, St. Louis, MO, USA) and either a polyclonal antiserotonin (raised in rabbit, diluted 1 : 500) (Sigma) or a polyclonal anti‐FMRFamide primary antibody (raised in rabbit, diluted 1 : 400) (Biotrend, Cologne, Germany) in 0.1 M PBS for 24 h at room temperature. This was followed by incubation in a reaction cocktail containing a goat anti‐mouse Alexa Fluor 488 and a goat anti‐rabbit Alexa Fluor 568 fluorescence‐coupled secondary antibody (dilution 1 : 300; Life Technologies, Vienna, Austria) and DAPI (dilution 1 : 200) in 0.1 M PBS in the dark for 24 h at room temperature. Finally, the specimens were rinsed in PBS and mounted on glass slides as described above. Negative controls were performed by incubating specimens of each developmental stage without either the primary or the secondary antibodies and rendered no specific signal.

### Analysis and digital image acquisition

Stained larvae and juveniles were analysed with a Leica TCS SP5 II confocal laser scanner (cLSM) mounted on a DM 6000 CS inverted microscope (Leica Microsystems, Wetzlar, Germany). Stacks of optical sections between 0.7 and 0.2 *μ*m thicknesses were generated and digitally merged into maximum projections. In‐depth analyses of the confocal stacks as whole‐mounts or as individual optical sections were performed using the 3D‐reconstruction software imaris 7.3 (Bitplane, Zürich, Switzerland). Finally, contrast and brightness of images was adjusted and figure plates were created with photoshop CS5 (Adobe Systems, San Jose, CA, USA). Schematic drawings were generated with corel draw 11.0 (Corel Corporation, Ottawa, Ontario, Canada).

## Results

### Neurogenesis: serotonin‐like immunoreactivity

**Figure 2:**
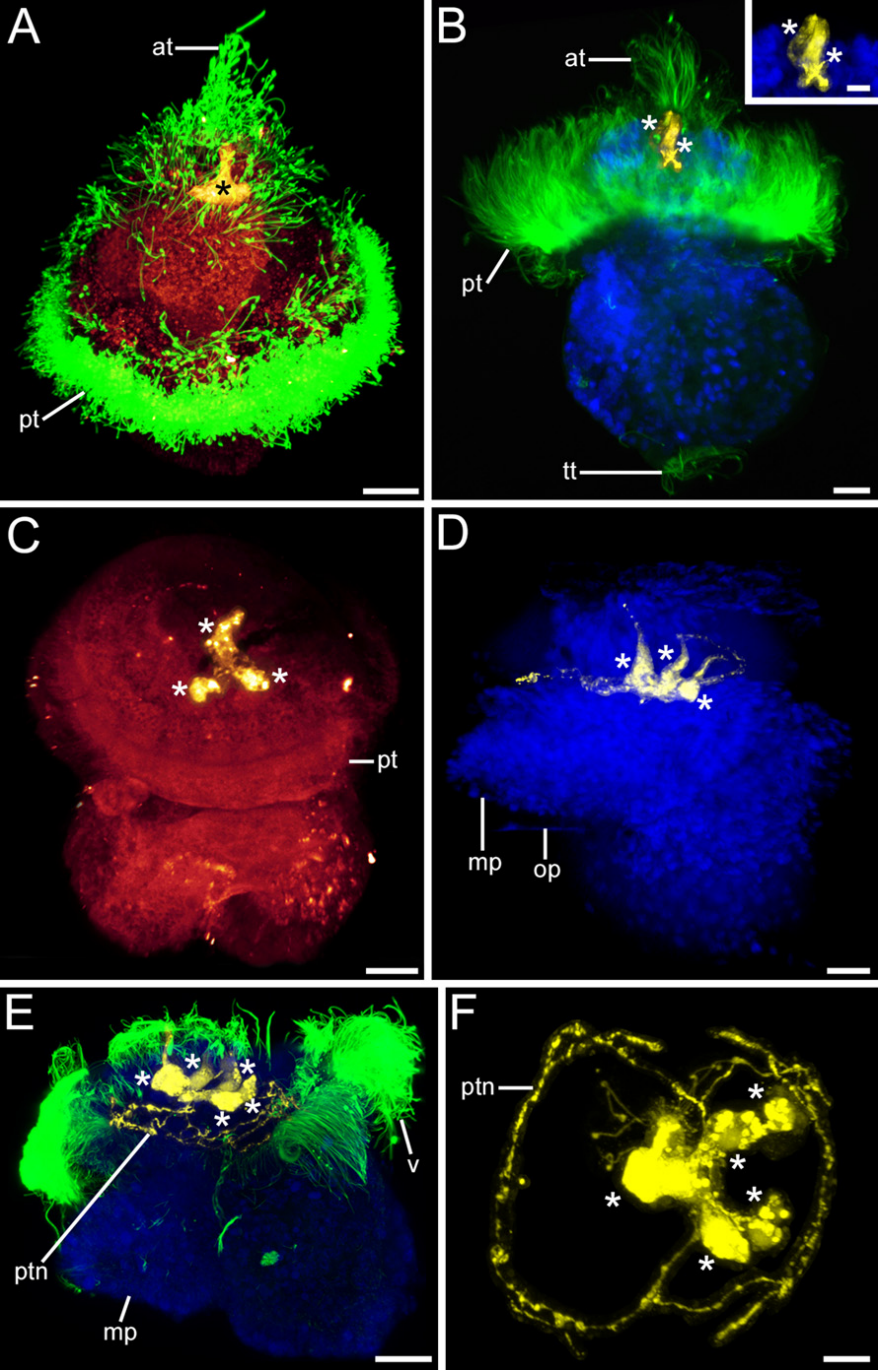
Serotonin‐like immunoreactivity (lir) in *Lottia* cf. *kogamogai* larvae. Serotonin‐lir, yellow; cell nuclei, blue; cilia, green. Anterior faces upwards in all images, except in (F) where it faces the viewing plane. (A‐C) dorso‐ventral view; (D‐E) lateral view with ventral to the left; (F) apical view with ventral to the left. Age of larvae is given in hours postfertilization (hpf). (A) trochophore larva (8 hpf) showing the apical organ with apical ciliary tuft (at) and a median, flask‐shaped cell (asterisk). pt, prototroch. (B) Slightly older larva (14 hpf) with two flask‐shaped cells in the apical organ. tt, telotroch. Inset magnification of the two flask‐shaped, apical cells. (C) veliger larva (20 hpf) with three, one median and two lateral, serotonin‐lir cells in the apical organ. (D) same stage larva as in (C) (20 hpf) showing that the three apical cells are flask‐shaped. mp, metapodium; op, operculum. (E) pediveliger larva (25 hpf) with five cells in the apical organ and a neurite ring (ptn) that underlies the ciliated prototroch. (F) same larva as in (E); apical organ that comprises one median and two lateral flask‐shaped as well as two round cells and from which neurites run to the prototroch and form a neurite ring. (A‐F) CLSM, maximum projections. Scale bars: (A‐E) = 20 *μ*m; inset, (F) = 10 *μ*m

**Figure 3:**
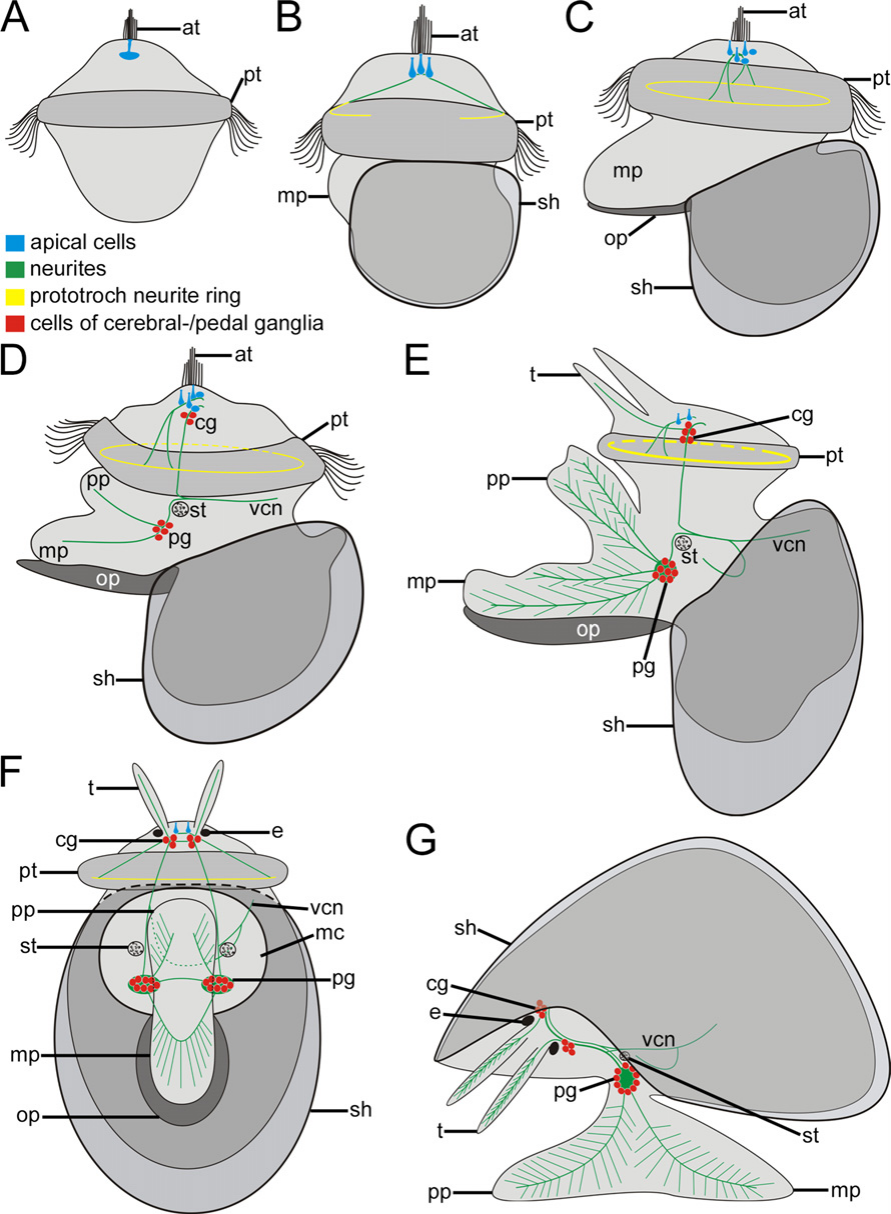
Schematic representation of serotonin‐like immunoreactivity (lir) in *Lottia* cf. *kogamogai*. Anterior faces upwards in all images, except in (G) where it faces to the left. (A) dorso‐ventral view; (B‐E) lateral view, ventral to the left; (F) ventral view; (G) lateral view, ventral facing down. Total size of specimens is approx. 110 *μ*m in (A); 120 *μ*m in (B); 125 *μ*m in (C); 140 *μ*m in (D); 170 *μ*m in (E, F); 200 *μ*m in (G). Age of larvae is given in hours or days postfertilization (hpf, dpf). (A) trochophore larva (8 hpf) with one median flask‐shaped cell beneath the apical tuft (at) within the apical organ. pt, prototroch. (B) early veliger larva (18 hpf) with three flask‐shaped apical cells, from which a neurite projects to the prototroch. mp, metapodium; sh, shell. (C) veliger larva (25 hpf), five apical cells, one median, two lateral flask‐shaped and two round cells. Note that the prototroch neurite forms a ring. op, operculum. (d) pediveliger larva (32 hpf), first cells of the future cerebral and pedal ganglia (cg, pg) appear. The meta‐ and propodium (mp, pp) of the emerging foot bears neurites that originate at the pedal ganglia. Note the statocyst (st) close to the pedal ganglia. vcn, visceral neurite. (E) late pediveliger larva (2 dpf), disintegration of the apical organ with now only two cells, elaborated nervous system with numerous cells in the cerebral and pedal ganglia. Neurites from the pedal ganglia form an extensive branching network within the foot, while the tentacles (t) exhibit neurites that originate at the cerebral ganglia. (F) same specimen as in (E) showing the nervous system of the peidveliger larva. Each cerebral ganglion is connected to the ipsilateral pedal ganglion by a connective while the contralateral cerebral or pedal ganglia are connected with each other by a commissure. Neurites from each cerebral ganglion now form the neurite that runs towards the visceral mass. e, eye; mc, mantle cavity. (G) early juvenile (7 dpf) prototroch, prototroch neurite ring, apical cells and operculum are lost. The nervous system labelled with antibodies to serotonin comprises the paired cerebral and pedal ganglia, from which neurites project into the tentacles and the foot, forming a complex neurite network.

**Figure 4:**
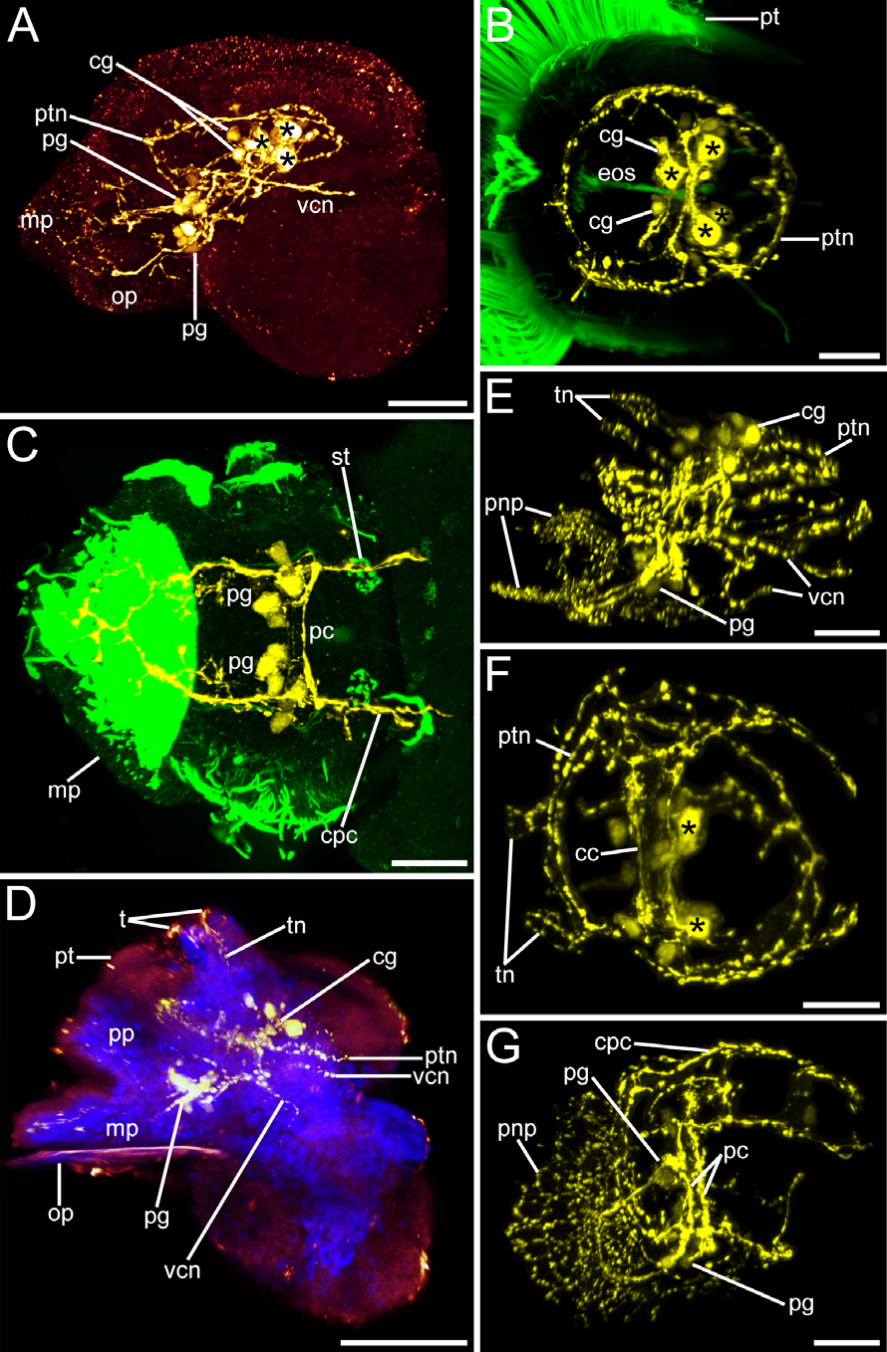
Serotonin‐like immunoreactivity (lir) in *Lottia* cf. *kogamogai* larvae. Serotonin‐lir, yellow; cell nuclei, blue; cilia, green. Anterior faces upwards in all images, except in (B, C, F) where it faces the viewing plane. (A, D, E, G) lateral view, ventral to the left; (B, C, F) apical view with ventral to the left. Age of larvae is given in hours or days postfertilization (hpf, dpf). (A) pediveliger larva (32 hpf), at the base of the apical organ (apical cells, asterisks) first cells of the adult cerebral ganglia (cg) are formed. Neurites run from the cerebral ganglia towards the pedal ganglia (pg), which are at the base of the foot and from which neurites project into the foot (mp). First visceral neurite (vcn) runs from the left cerebral ganglion towards the visceral mass. op, operculum; ptn, prototroch neurite ring. (B) same specimen as in (A) showing the anterior part of the larva only. First cells of the cerebral ganglion appear beneath the apical organ that comprises five cells of which only four are visible. oes, oesophagus; pt, prototroch. (C) same specimen as in (A) showing the posterior part of the larva only. The pedal ganglia project neurites into the foot, are connected to each other by pedal commissures (pc) and with the cerebral ganglia by the cerebropedal connectives (cpc). The statocysts (st) appear posterior to the pedal ganglia. (D) late pediveliger larva (2 dpf) showing neurites (tn) running from the cerebral ganglia towards the developing tentacles (t), while the meta‐ and propodium of the foot exhibit neurites that originate at the pedal ganglia. (E) same stage larva as in (D) showing the elaborated nervous system labelled with antibodies to serotonin with an extensive branching neurite network within the larval foot (pnp). (F) same specimen as in (E) showing the anterior part only. The apical organ comprises only two cells now, while neurites from the cerebral ganglia run into the tentacles. cc, cerebral commissure. (G) same specimen as in (E) showing the posterior part only. The extensive branching neurite plexus in the larval foot is derived from the pedal ganglia. (A‐G) CLSM, maximum projections. Scale bars: (A, D) = 50 *μ*m; (C‐B, E‐G) = 20 *μ*m

**Figure 5:**
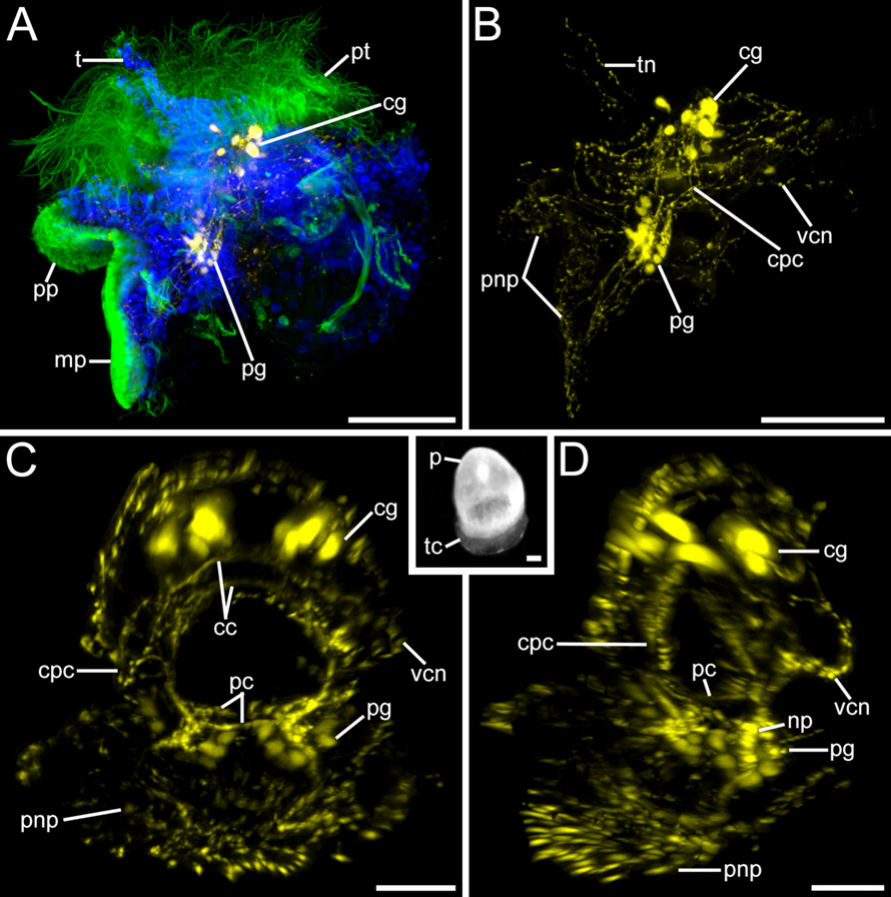
Serotonin‐like immunoreactivity (lir) in *Lottia* cf. *kogamogai* larvae. Serotonin‐lir, yellow; cell nuclei, blue; cilia, green. Anterior faces upwards in all images, except inset where it faces downwards. (A‐B, D) lateral view, ventral to the left; inset in (C), dorsal view. Age of larvae is given in days postfertilization (dpf). (A) metamorphic competent larva (3 dpf) with elaborated cerebral (cg) and pedal ganglia (pg) and no labelled cells within the apical organ. mp, metapodium; pp, propodium; t, tentacle; pt, prototroch. (B) same larva as in (A) showing the entire nervous system labelled with antibodies to serotonin. cpc, cerebropedal connective; pnp, pedal neurite complex; tn, tentacle neurite; vcn, visceral neurite. (C) metamorphosis (5 dpf). Specimen showing the paired cerebral and pedal ganglia which are connected to each other by commissures (cc, pc) and connectives. Inset, same stage as in (C) future adult shell (teleoconch, tc) begins to develop at the base of the protoconch (pt). (D) same specimen as in (C) pedal ganglia relatively large with numerous cells in each ganglion arranged on the outside, while their neurites form a neuropil (np) on the inside. (A‐C, D) CLSM, maximum projections. Inset, stereo micrograph. Scale bars: (A, B, inset) = 50 *μ*m; (C, D) = 20 *μ*m

In *Lottia* cf. *kogamogai*, as early as in 8 hpf old trophophore larvae, the first detectable serotonin‐like immunoreactivity (lir) signal appears in a flask‐shaped cell that is located in the anterior region below the apical tuft, that is the apical organ (Figs 2A and 3A). As development proceeds, a second (14 hpf) and a third serotonin‐lir flask‐shaped cell appear (veliger, 20 hpf) in the apical organ of the larva, where they flank the first serotonin‐positive cell (Figs 2B–D and 3B). In the pediveliger larva (25 hpf) two round cells appear slightly posterior to the flask‐shaped cells and a neurite ring that underlies the cilia‐bearing prototroch (Figs 2E, F and 3C). During subsequent development serotonin‐lir cells appear at the base of the apical organ and the foot. These cells are part of the paired *Anlagen* of the future adult cerebral and pedal ganglia, respectively (32 hpf) (Figs 3D and 4A–C). Cerebral and pedal ganglia *Anlagen* are connected with each other by connectives. Each cerebral and pedal hemiganglion is also connected by commissures respectively (Figs 3D and 4A–C). In addition, a right and left statocyst proximal to the pedal ganglia is visible at this stage (Figs 3D and 4C). Above one of the statocysts, specifically the left one, a neurite runs towards the visceral mass between the (left) cerebral and pedal ganglion *Anlagen* (Figs 3D and 4A). At the same time, numerous neurites from the pedal ganglia *Anlagen* run into the anterior (propodium) and posterior (metapodium) portion of the growing foot (Figs 3D and 4A, C). After 2 dpf eyes and tentacles have developed and the serotonin‐lir nervous system has elaborated considerably. The number of cells in the forming ganglia increases. From the cerebral ganglia neurites project in each tentacle and into the visceral mass (Figs 3E,F and 4D–G). The branching network of neurites (i.e. neurite plexus) in the foot originates in the pedal ganglia (Figs 3E,F and 4D–G). Interestingly, at this stage, the apical organ seems to start disintegrating as it shows only two of the five serotonergic cells (Figs 3E,F and 4F). Taken from the position of the two remaining flask‐shaped apical cells, it seems that the central flask‐shaped and the two round cells have disappeared or at least have ceased to express serotonin (Figs 3E,F and 4F). Towards metamorphosis (3–5 dpf), larvae, now with long tentacles which are supplied by neurites from the cerebral ganglia, increase in size and show a prominent foot with an extensive neurite plexus and relatively large pedal ganglia containing numerous serotonin‐lir cells and a neuropil (Fig. 5A,B). At this stage no serotonin‐positive apical cell is detectable (Fig. 5A,B). At metamorphosis (5–7 dpf), the teleoconch starts to form, while the larval operculum as well as the neurite ring that underlies the prototroch and its cilia are lost (Figs 3G and 5C,D).

### Neurogenesis: FMRFamide‐like immunoreactivity

**Figure 6:**
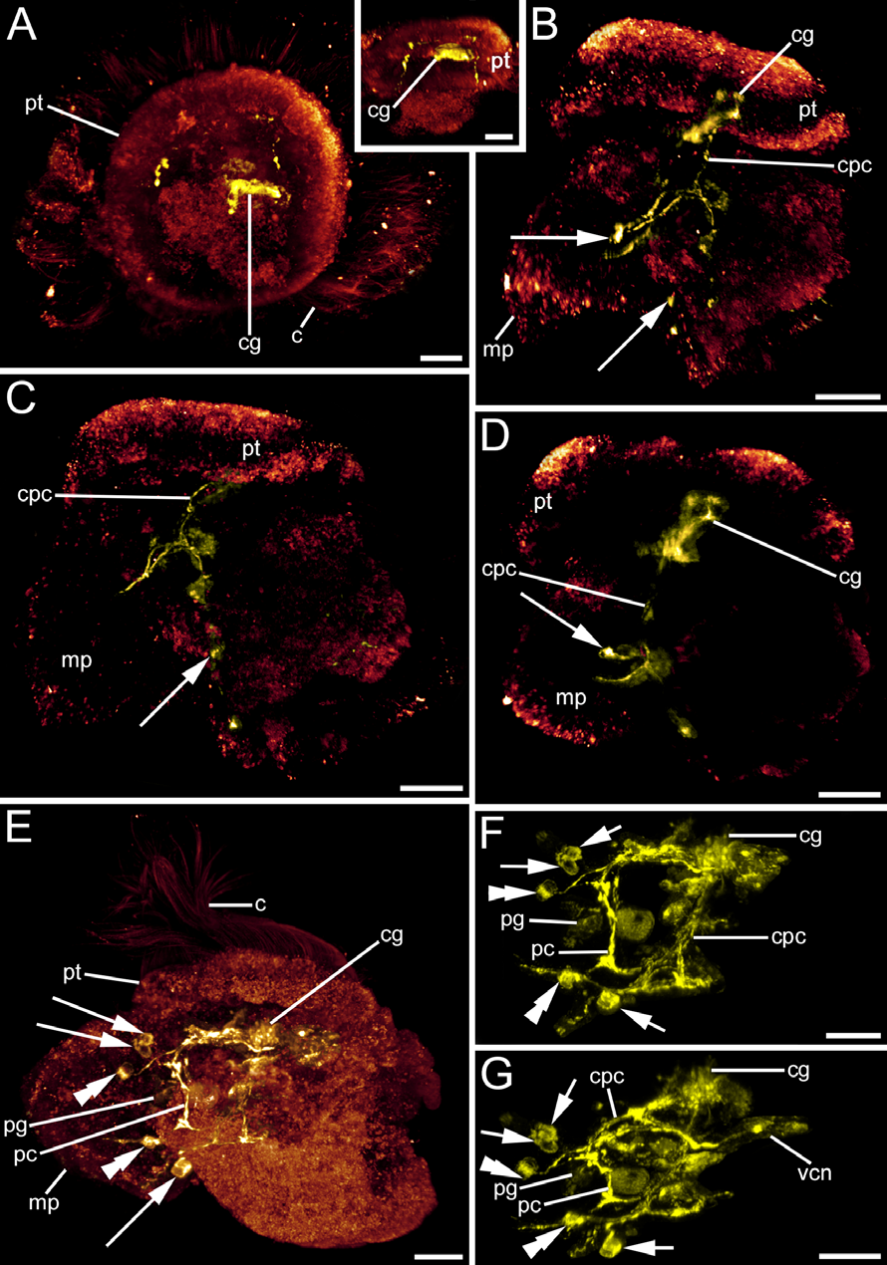
FMRFamid‐like immunoreactivity (lir) in *Lottia* cf. *kogamogai* larvae. Anterior faces upwards in all images, except in (A) where anterior faces the viewing plane. (A) apical view; inset, (B‐G) lateral view, ventral to the left. Age of larvae is given in hours postfertilization (hpf). (A) late veliger larva (24 hpf) with first FMRFamide‐positive signal in the cerebral ganglia (cg) *Anlagen*. c, cilia; pt, prototroch. Inset, a pair of neurites runs from the cerebral ganglia towards the mid‐body region. (B) slightly older larva as in (A) (25 hpf) showing the paired neurites, which will form cerebropedal connectives (cpc) that bifurcate in the mid‐body region with one neurite running towards the growing metapodium (mp) and one neurite that runs towards the base of the foot. There, one flask‐shaped cell on each lateral side is visible (arrows). (C) same larva as in (B) showing the left side only. (D) same larva as in (B) showing the right side only. Note the flask‐shaped cell at the base of the metapodium. (E) pediveliger larva (32 hpf) with a nervous system labelled with antibodies to FMRFamide that comprises the cerebral and pedal ganglia. In contrast to the cerebral ganglia, numerous cells are visible in the pedal ganglia, which are connected to each other by a commissure (pc). From each pedal ganglion neurites run into the metapodium, in which bipolar cells are visible (double arrowheads). At the base of the foot and close to each pedal ganglion a pair of flask‐shaped cells is visible. (F) same larva as in (E) showing the entire nervous system labelled with antibodies to FMRFamide. Each cerebropedal connective bifurcates in the mid‐body region and one neurite runs to the respective pedal ganglion, while the other neurite runs to the base of the metapodium where one pair of flask‐shaped cells is located. (G) same larva as in (E) but slightly tilted upwards. Note that a neurite splits of the left cerebropedal connective and runs towards the visceral mass (vcn). (A‐G) CLSM, maximum projections. Scale bars: (A‐G) = 20 *μ*m

**Figure 7:**
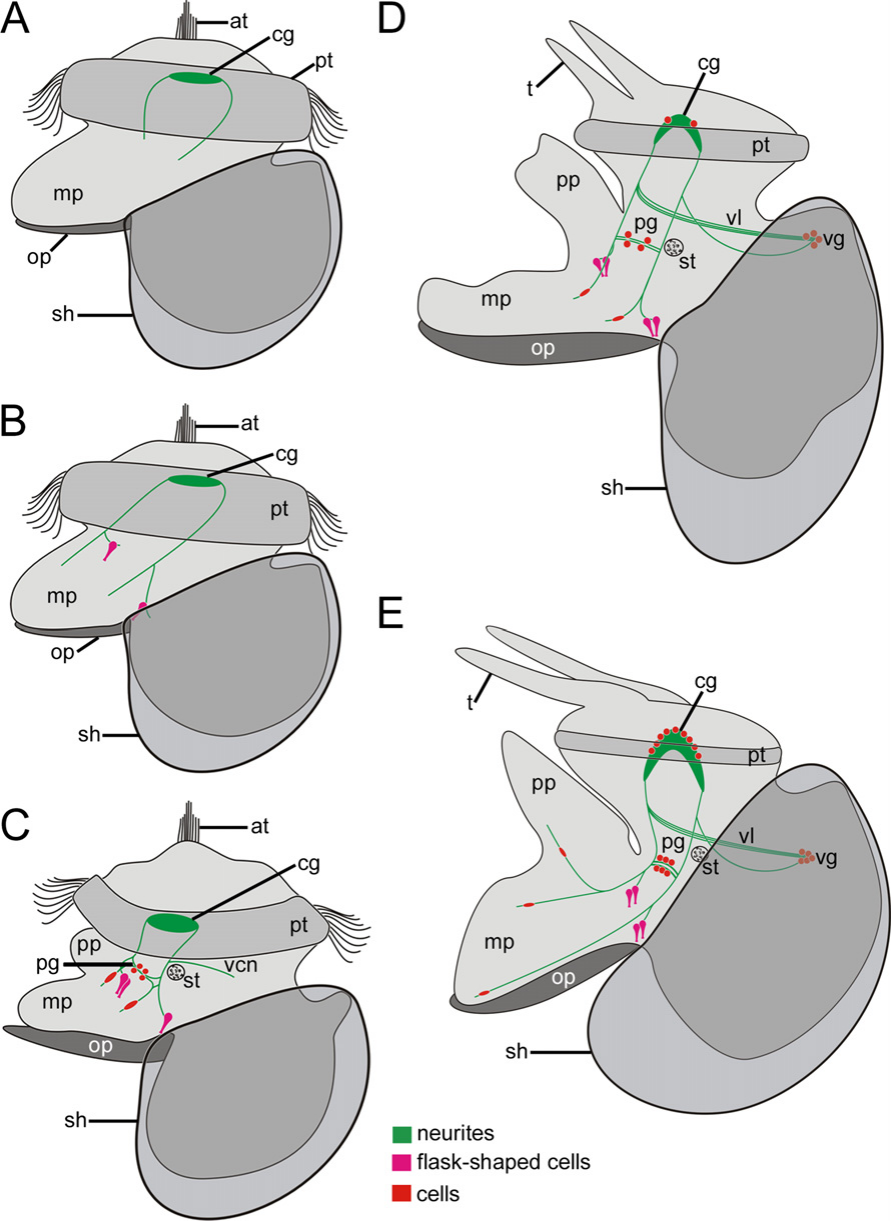
Schematic representation of FMRFamide‐like immunoreactivity (lir) in *Lottia* cf. *kogamogai*. Anterior faces upwards in all images. (A‐E) lateral view, ventral to the left. Total size of specimens is approx. 120 *μ*m in (A); 125 *μ*m in (B); 140 *μ*m in (C); 170 *μ*m in (D); 180 *μ*m in (E). Age of larvae is given in hours or days postfertilization (hpf, dpf). (A) neurites are the first labelled neuronal structures that appear in the future adult cerebral ganglia and cerebropedal connectives in the late veliger larva (24 hpf). at, apical tuft; mp, metapodium; op, operculum; pt, prototroch; sh, shell. (B) slightly older larva as in A (25 hpf) showing flask‐shaped cells at the base on each side of the emerging foot. Note that the paired neurites, *Anlage* of the cerebropedal connectives, bifurcate in the mid‐body region. (C) pediveliger larva (32 hpf) with cells within the pedal ganglia (pg) *Anlagen* as well as within the foot. Note the first visceral neurite (vcn) close to the statocyst (st) that runs posteriodorsally towards the visceral mass. pp, propodim. (D) late pediveliger larva (2 dpf) with first labelled cells within the cerebral ganglia and the visceral ganglion (vg). Note that the visceral loop (vl) is labelled as well. t, tentacle. (E) metamorphic competent larva (3 dpf) with a more elaborated nervous system labelled with antibodies to FMRFamide. The number of cells increases in all ganglia (cerebral, pedal, visceral) as well as within the foot, which by this time exhibits numerous neurites.

**Figure 8:**
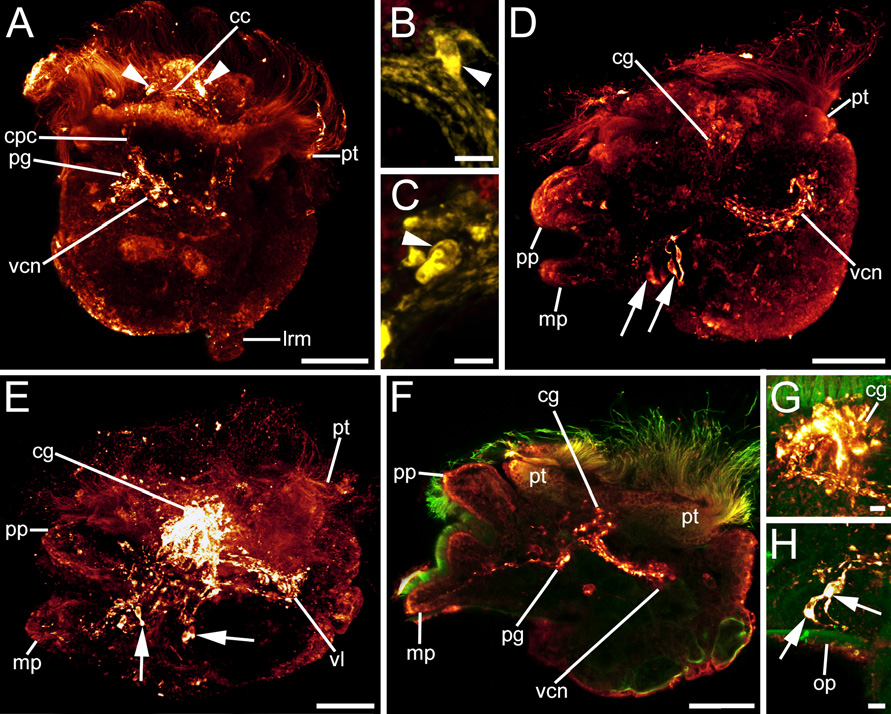
FMRFamid‐like immunoreactivity (lir) in *Lottia* cf. *kogamogai* larvae. FMRFamide‐lir, red A, D–H or yellow B, C; cilia, green. Anterior faces upwards in all aspects. A–C dorsal view; D–H lateral view, ventral to the left. Age of larvae is given in days postfertilization (dpf). A late pediveliger larva (2 dpf), cerebral commissure (cc) between the future cerebral ganglia and with first labelled cells (arrowheads) in each ganglion visible. cpc, cerebropedal connective; lrm, main larval retractor muscle; pg, pedal ganglion; pt, prototroch; vcn, visceral neurite. B same larva as in A magnification of the cell in the right cerebral ganglion *anlage*. C same larva as in A magnification of the cell in the left cerebral ganglion *anlage*. D same stage larva as in A. Note the paired flask‐shaped cells (arrows) on each side at the base of the foot and the numerous neurites (vcn) that run from the cerebral ganglia (cg) posteriorly towards the visceral mass. mp, metapodium; pp, propodium. E metamorphic competent larva (3 dpf) with an elaborate nervous system labelled with antibodies to FMRFamide that is composed of strongly stained cerebral ganglia, from which neurites run posteriorly towards the visceral mass forming the visceral loop (vl). F same stage larva as in E optical section showing part of a cerebral and pedal ganglion (pg) from which neurites run towards the visceral mass and into the foot. G same larva as in F magnification of the relatively large future adult, horseshoe‐shaped cerebral ganglia. H same larva as in F magnification of one pair of flask‐shaped cells at the base of the foot. op, operculum. A–H CLSM, maximum projections, except F, which is an optical section. Scale bars: A, D, E, F = 30 *μ*m; B, C, G–H = 5 *μ*m

The earliest FMRFamide‐like immunoreactivity (lir) appears in late veliger stage larvae (24 hpf), mainly in neurites of the future cerebral ganglia, from which slightly later (25 hpf) paired lateral neurites, the *Anlage* of the cerebropedal connectives, are formed (Figs 6A–D and 7A,B). In addition, one neurite branches off in the mid‐body region from each lateral neurite and runs towards one flask‐shaped cell at the base of the foot (Figs 6B–D and 7B). As in the serotonin‐lir expression pattern, the *Anlagen* of the cerebral and pedal ganglia as well as an additional visceral neurite are clearly visible at a stage when the propodium starts to form (pediveliger stage, 32 hpf) (Figs 6E–G and 7C). While there are only neurites and no FMRFamide‐positive cells detectable in the forming cerebral ganglia, the pedal commissure and a few FMRFamide‐lir cells are visible in the pedal ganglion *Anlagen* (Figs 6E–G and 7C). Moreover, and unlike the serotonin‐lir pattern, cells containing FMRFamide neuropeptides are not only present in the ganglia but also in the periphery. Namely, there are flask‐shaped cells at the base of the foot as well as bipolar cells within the foot (Figs 6E–G and 7C). During subsequent development the FMRFamide‐lir nervous system elaborates dramatically. Notably, numerous neurites form the visceral loop, the pedal commissures, and the cerebropedal connectives. Also, FMRFamide‐lir cells start to appear in the cerebral ganglia and along the visceral neurites as well as in the *Anlage* of the visceral ganglion, which is located dorso‐posteriorly to the cerebral ganglia (2 dpf) (Figs 7D and 8A–D). Towards metamorphosis (3 dpf), the number of FMRFamide‐lir cells and neurites that form the now horseshoe‐shaped cerebral ganglia increases dramatically (Figs 7E and 8E,G). In addition, the number of FMRFamide‐positive cells in the pedal ganglia and in the visceral ganglion increases, while the number of flask‐shaped cells at the base of the foot on each side remains constant (Figs 7E and 8E–H). The tentacles show no sign of FMRFamide‐like neuropeptides, and neurites within the foot do not form a complex branching network, which is unlike the serotonin‐lir pattern (Figs 7D,E and 8A,D,E,F).

### Myogenesis

**Figure 9:**
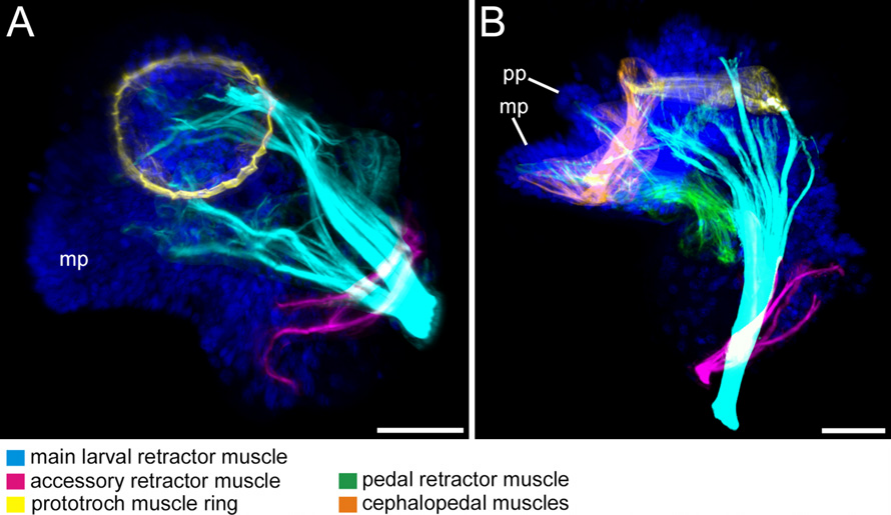
Musculature of *Lottia* cf. *kogamogai* larvae. Anterior faces upwards in all images. A, B lateral view, ventral to the left. Age of larvae is given in hours postfertilization (hpf). A late veliger larva (24 hpf) with two, larval main and accessory, retractor muscles and a prototroch muscle ring. Note that the accessory retractor muscle inserts dorso‐anteriorly to the insertion site of the main larval retractor muscle. B pediveliger larva (32 hpf), the retractor muscles are now more ventrally due to the process of torsion, while two new muscles appear, the pedal retractor and the herein newly discovered, paired cephalopedal muscles. A, B CLSM, maximum projections. Scale bars: A, B = 30 *μ*m

**Figure 10:**
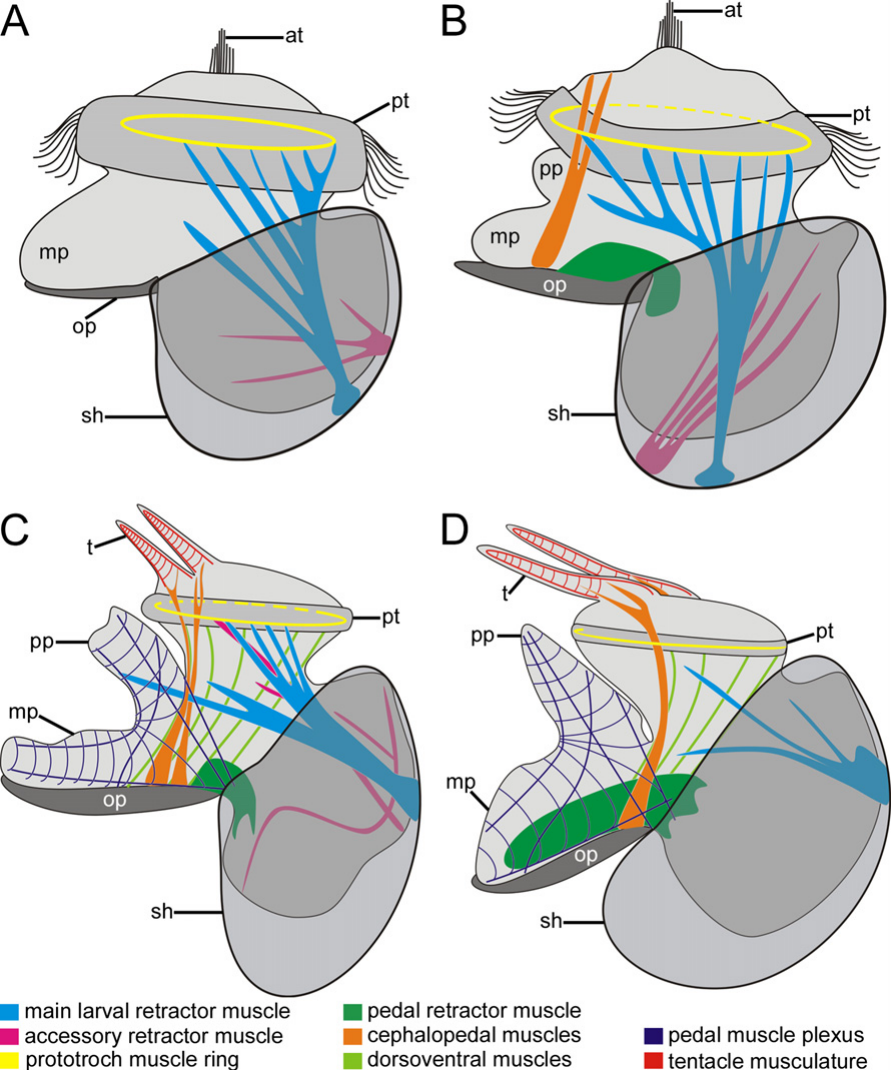
Schematic representation of myogenesis in *Lottia* cf. *kogamogai*. Anterior faces upwards in all aspects. (A‐D) lateral view, ventral to the left. Total size of specimens is approx. 120 *μ*m in (A); 140 *μ*m in (B); 170 *μ*m in (C); 180 *μ*m in (D). Age of larvae is given in hours or days postfertilization (hpf, dpf). (A) late veliger larva (20 hpf) exhibits two retractors, the larval main and accessory muscle, and a prototroch muscle ring. at, apical tuft; mp, metapodium; op, operculum; pt, prototroch; sh, shell. (B) post‐torsional pediveliger larva (32 hpf) showing two newly formed muscles, the paired cephalopedal muscles (described for the first time herein) and the pedal retractor muscle. Note that due to the process of torsion the retractor muscles now occupy a more ventral position with respect to the pretorted condition. pp, propodium. (C) late pediveliger larva (2 dpf) with a meshwork of longitudinal and transverse muscle fibres in the foot and the tentacles (t) and dorso‐ventral muscle fibres that run from the dorsal region of the head and visceral mass towards the foot. Note that the ventral branch of the main larval retractor muscle runs to the anterior part of the foot, while a branch of the accessory retractor muscle runs to the velum. (D) metamorphic competent larva (3 dpf), increase in size in the pedal retractor muscle and decrease in the prototroch muscle ring and the larval retractor muscle, while the accessory retractor muscle is no longer visible (disintegration process)

**Figure 11:**
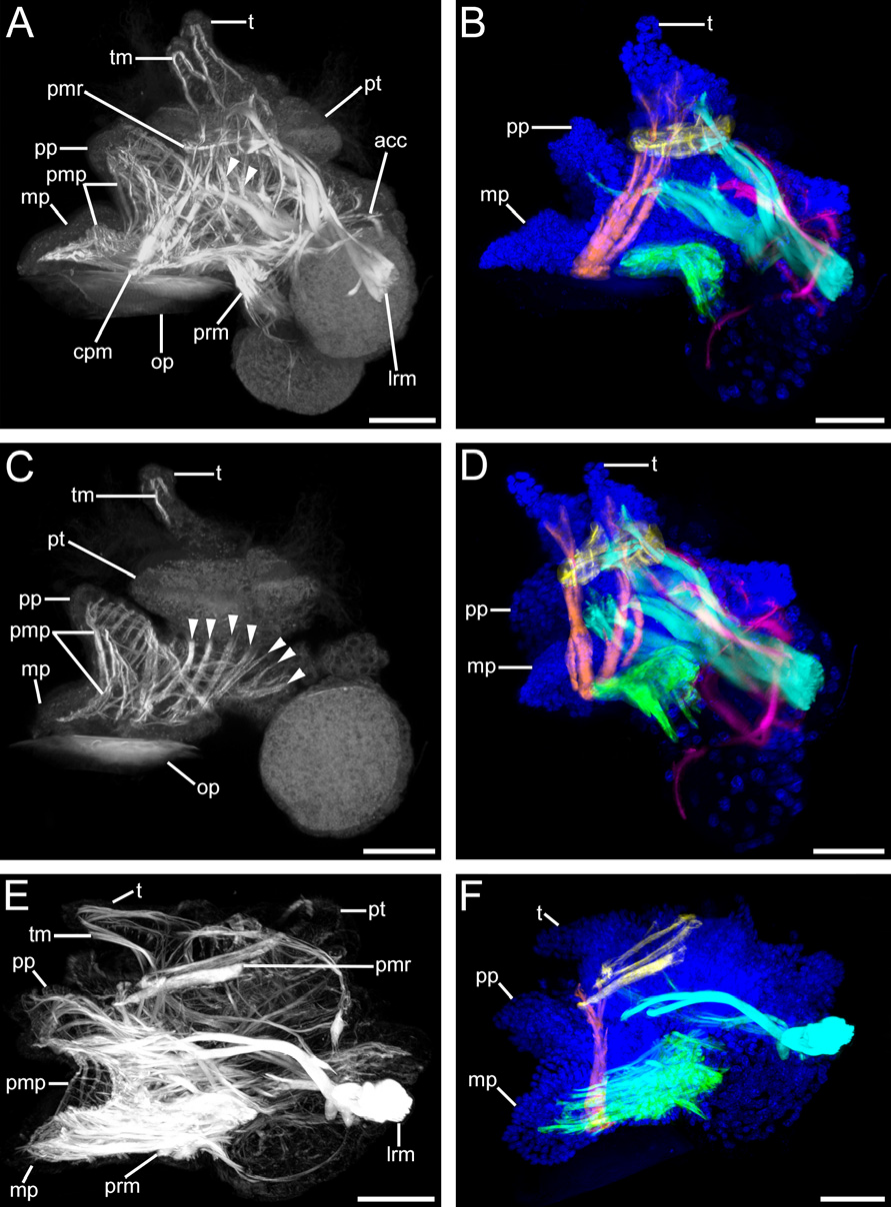
Musculature of *Lottia* cf. *kogamogai* larvae. (A, C, E) musculature in white; (B, D, F) muscles colour coded: blue, cell nuclei; cyan, main larval retractor muscle; green, pedal retractor muscle; orange, cephalopedal muscle; pink, accessory retractor muscle; yellow, prototroch muscle ring. (B, D, F) the tentacle, foot and dorso‐ventral musculature are omitted for clarity. Anterior faces upwards in all images. (A‐F) lateral view, ventral to the left. Age of larvae is given in days postfertilization (dpf). (A) late pediveliger larva (2 dpf) showing a prominent main larval retractor muscle (lrm) that runs from the insertion site anteriorly into the prototroch (pt) and the propodium (pp), while branches of the accessory retractor muscles (acc) run along the mantle and to the prototroch. The foot and the tentacles (t) comprise a meshwork of longitudinal and transversal muscle fibres (pmp, tm, respectively). Note the dorso‐ventral muscle fibres (arrowheads) that run from the head and visceral mass towards the foot. cpm, cephalopedal musculature; mp, metapodium; op, operculum; pmr, prototroch muscle ring; prm, pedal retractor muscle. (B) same larva as in (A) showing main muscle systems. Note that the paired cephalopedal muscle inserts at the base of the metapodium and runs towards the base of the respective ipsilateral tentacle. (C) same specimen as in (A), optical section showing the muscular meshwork within the larval foot and numerous dorso‐ventral muscles. (D) same specimen as in (A), slightly tilted to the left, the paired nature of the cephalopedal musculature is visible, which runs through the head, crossing the prototroch muscle ring on the inner side, to each ipsilateral base of the tentacle. Note that the ventral branch of the main larval retractor muscle runs into the more anterior part of the foot, the propodium. (E) metamorphic competent larva (3 dpf) with a dominant pedal retractor muscle, while the accessory retractor muscle is no longer visible and the prototroch muscle ring as well as the main larval retractor muscle are smaller than in the stage before. (F) same specimen as in (E) the prototroch muscle ring and the main larval retractor muscle show signs of disintegration. (A‐F) CLSM, maximum projections, except (C), which is an optical section. Scale bars: (A‐F) = 30 *μ*m

Two larval retractors, that is, the main and accessory (i.e. mantle) retractor muscle, as well as the prototroch muscle ring appear as the first muscular structures in the late veliger larva (24 hpf) (Figs 9a and 10a). Both retractor muscles insert dorso‐posteriorly on the inner wall of the protoconch, from where several branches split off and run towards the prototroch, foot, and mantle (Figs 9A and 10A). Due to the process of torsion, a 180° rotation of the visceral body (visceropallium) relative to the head and foot (cephalopodium) in the pediveliger larva (32 hpf), both larval retractors have a more ventral location with respect to the pretorsional cephalopodium (Figs 9A,B and 10A,B). Notably, in the post‐torsional larva the position of the accessory larval retractor muscle has changed from a dorsal to a ventro‐median position (Figs 9A,B and 10A,B). At the same time a prominent pedal retractor muscle appears ventro‐anteriorly to the accessory retractor muscle and lances the metapodium (Figs 9B and 10B). In addition, one pair of newly discovered muscle bundles, here termed the cephalopedal muscles, project from the metapodium on each lateral side into the head region (Figs 9B and 10B). As the larvae grow (2 dpf), the main larval retractor becomes the predominant muscle with solid branches that run towards the head and the propodium (anterior part of the growing foot), while branches of the accessory retractor muscle run along the mantle and towards the velum (Figs 10C and 11A–D). Pediveliger larvae at this stage exhibit well‐differentiated pedal and tentacular musculature nets that consist of longitudinal and transversal muscle fibres (Figs 10C and 11A,C). Moreover, dorso‐ventral muscle fibres run ventrally from the dorsal part of the head and the visceral mass towards the foot of the larva (Figs 10C and 11A,C). Towards metamorphosis, the larval retractor muscles and the velum muscle ring are either lost or in the process of disintegration, (Figs 10D and 11E,F). At the same time, other muscles such as the pedal muscle meshwork, the predominant pedal retractor, the paired cephalopedal as well as the dorso‐ventral and tentacular musculature elaborate (Figs 10D and 11E,F).

## Discussion

**Figure 12:**
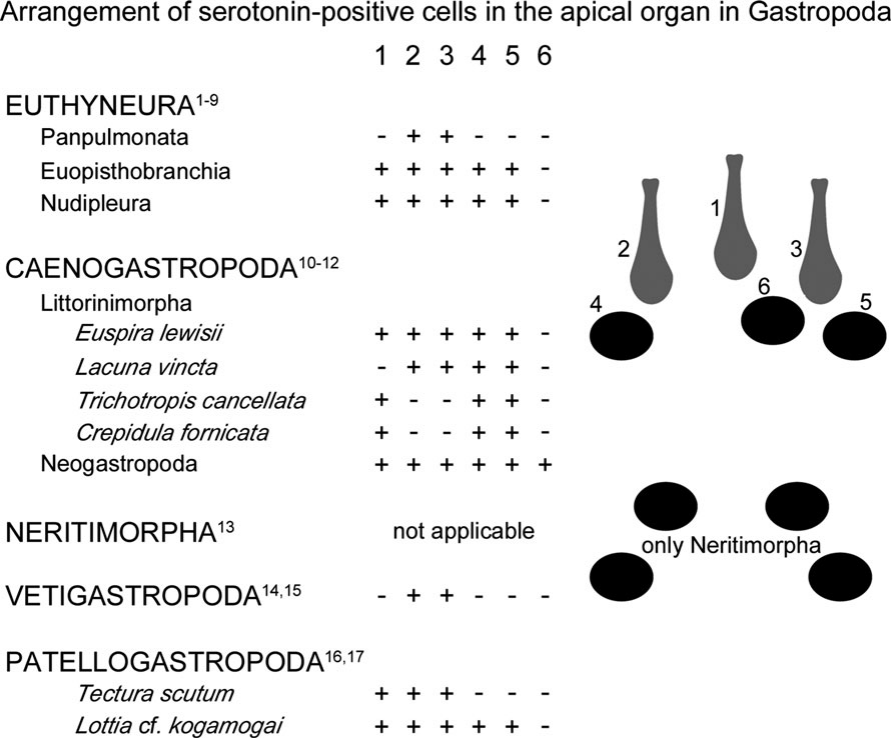
Arrangement of the serotonin‐lir cells within the apical organ in gastropod larvae. Anterior faces up in the drawings. Colour code: grey, flask‐shaped cells; black, round cells. The numbers 1–6 correspond to the apical cells in the upper drawing on the right side. 1, median flask‐shaped cell; 2, 3, lateral flask‐shaped cells; 4–6, round cells. +, present; −, absent. The lower drawing on the right side shows the different arrangement of serotonin‐positive cells within the apical organ in Neritimorpha. Classification after Schrödl (2014). References: 1, Diefenbach et al. 1998; 2, Glebov et al. 2014; 3, Marois and Carew 1997a; 4, Dickinson et al. 2000; 5, Kempf et al. 1997; 6, LaForge and Page 2007; 7, Kristof and Klussmann‐Kolb 2010; 8, Ruiz‐Jones and Hadfield 2011; 9, Kempf and Page 2005; 10, Page and Parries 2000; 11, Dickinson et al. 1999; 12, Dickinson and Croll 2003; 13, Page and Kempf 2009; 14, Barlow and Truman 1992; 15, Page 2006a; 16, Page 2002a; 17, present study

**Figure 13:**
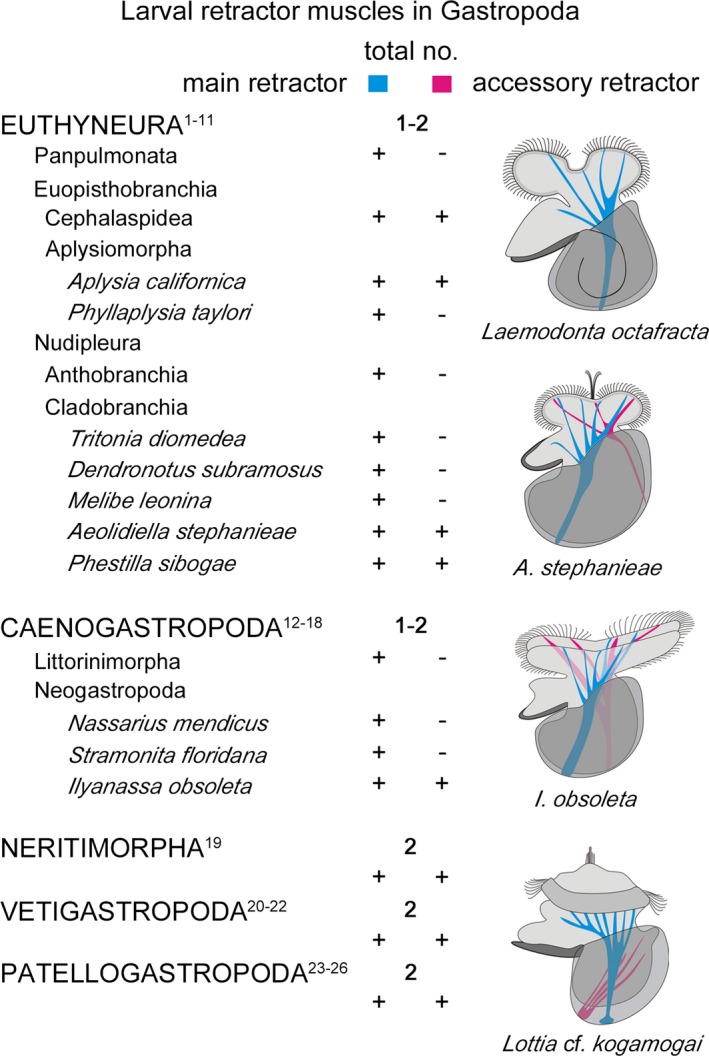
Larval retractor muscles in gastropod larvae. Anterior faces up in the drawings, which illustrate the different muscular situations present in Gastropoda. Numbers indicate the amount of retractor muscles within a clade (e.g. Panpulmonata) while ‘+’ marks present and ‘–’ absent. Classification after Schrödl (2014). References: 1, Ruthensteiner and Schaefer 2002; 2, Kawaguti and Yamasu 1960a; 3, Kawaguti and Yamasu 1960b; 4, Smith 1967; 5, Horikoshi 1967; 6, Wollesen et al. 2008; 7, Bridges 1975; 8, Page 1995; 9, Thompson 1958; 10, Kristof and Klussmann‐Kolb 2010; 11, Bonar and Hadfield 1974; 12, Werner 1955; 13, Fretter 1972; 14, D'Asaro 1965; 15, Page 1998; 16, Page 2006b; 17, D'Asaro 1966; 18, Evans et al. 2009; 19, Page and Ferguson 2013; 20, Degnan et al. 1997; 21, Page 1997; 22, Page 2002b; 23, Wanninger et al. 1999a; 24, Damen and Dictus 2002; 25, Smith 1935; 26, present study

Information on nervous and/or muscle system development is available for representatives from all five major gastropod clades (Patello‐, Veti‐, Caenogastropoda, Neritimorpha, Heterobranchia; Fig. 1) (e.g. Barlow and Truman 1992; Wanninger et al. 1999a; Croll 2000; Page and Parries 2000; Page 2002a,b, 2006a,b; Ruthensteiner and Schaefer 2002; Evans et al. 2009; Page and Kempf 2009; Kristof and Klussmann‐Kolb 2010; Page and Ferguson 2013). The data presented herein on the limpet *Lottia* cf. *kogamogai* revealed not only new structures but also structures known across gastropods. Accordingly, a comparative analysis allows for considerable insight into putative features of the ancestral gastropod larval bodyplan (Figs 12 and 13). Here, for the first time the nervous system labelled with antibodies against FMRFamide is characterized in patellogastropod larvae from early development until after metamorphosis, supplemented by data for the developing serotonin‐lir neural subsets and myogenesis.

### Early neurogenesis in Gastropoda

First neural structures immunoreactive to serotonin have often been reported in the apical organ (also described as apical or cephalic sensory organ) in indirect and some direct developing gastropods (reviewed by Croll and Dickinson 2004). As species with direct development show only few or no serotonergic cells in the apical organ, it is assumed that the differences in number of apical cells correlate with the different life histories (see Croll and Dickinson 2004 and references therein). This is corroborated on the ultrastructural level by transmission electron microscope studies, in which heterobranch species with free‐swimming larvae possess numerous cells (11–20) within the apical organ, while no or a reduced number of cells are reported within the apical organ in direct developing heterobranchs (Bonar 1978; Chia and Koss 1984; Marois and Carew 1997a; Ruthensteiner and Schaefer 2002). Further insights come from caenogastropod and euthyneuran larvae, where the apical organ consists of different types of sensory cells (ampullary or flask‐shaped and para‐ampullary or round cells) of which almost a third may express the neurotransmitter serotonin (Bonar 1978; Chia and Koss 1984; Marois and Carew 1997b; Page and Parries 2000; Schaefer and Ruthensteiner 2001; LaForge and Page 2007). In *Lottia* cf. *kogamogai*, the apical organ exhibits one median, two lateral flask‐shaped, and two round serotonin‐lir cells. Interestingly, the same arrangement of serotonin‐lir cells within the apical organ has been reported in euopisthobranch and nudipleuran veliger larvae as well as in the caenogastropod *Euspira lewisii* (Fig. 12) (Kempf et al. 1997; Marois and Carew 1997a; Dickinson et al. 2000; Page and Parries 2000; LaForge and Page 2007; Kristof and Klussmann‐Kolb 2010; Ruiz‐Jones and Hadfield 2011). This arrangement of apical cells immunoreactive to serotonin varies in other caenogastropods, panpulmonates, vetigastropods, and the patellogastropod *Tectura scutum*, while in the neritimorph gastropod *Nerita melanotragus*, on the other hand, an entirely different pattern of four round serotonin‐lir cells is present (Fig. 12) (Barlow and Truman 1992; Diefenbach et al. 1998; Dickinson et al. 1999; Page and Parries 2000; Page 2002a, 2006a; Dickinson and Croll 2003; Page and Kempf 2009; Glebov et al. 2014). Several putative functions such as the control of locomotion, feeding, and settlement have been assigned to the apical organ in gastropod larvae (e.g. Mackie et al. 1976; Arkett et al. 1987; Marois and Carew 1997a,b,c; Hadfield et al. 2000; Hadfield and Koehl 2004). Congruent with these assumptions is that with the reduction of larval structures, such as the ciliated velar lobes in direct developing species (e.g. *Haliosoma trivolis*), fewer cells are found within the apical organ than in species with long‐lived larva and enlarged velar lobes (e.g. *Ilyanassa obsoleta*) (Diefenbach et al. 1998; Dickinson and Croll 2003; Glebov et al. 2014). However, some but not all of these differences in the pattern of serotonin‐lir apical cells can be explained by the different developmental modes (i.e. planktotrophic vs. lecithotrophic vs. direct development). For instance, a clear difference is seen on both, ultrastructural and immunohistochemical level in the number and organization of cells within the apical organ between direct developing and planktotrophic developing Panpulmonata, while the morphology of the apical organ in Euopisthobranchia and Nudipleura, regardless of the developmental mode, is almost invariant (Marois and Carew 1997a,b,c; Diefenbach et al. 1998; Voronezhskaya et al. 1999; Schaefer and Ruthensteiner 2001; Ruthensteiner and Schaefer 2002; Kempf and Page 2005; LaForge and Page 2007; Kristof and Klussmann‐Kolb 2010; Glebov et al. 2014). Furthermore, there is a difference in three versus five serotonergic cells between the patellogastropods *Tectura scutum* (Page 2002a) and the herein investigated *L*. cf. *kogamogai*, although both have a lecithotrophic development of similar length and a relatively small, undivided ciliated prototroch.

An apical organ with serotonin‐lir flask‐shaped cells is not only commonly found across molluscan but also other spiralian/lophotrochozoan larvae (Friedrich et al. 2002; Voronezhskaya et al. 2002, 2008; Wanninger and Haszprunar 2003; Dyachuk and Odintsova 2009; Wanninger 2009, 2015a,b; Redl et al. 2014). Hence, a possible explanation might be that the serotonin‐lir five‐celled pattern, which is unique among molluscs (and other lophotrochozoans) and consists of two round, one median and two lateral flask‐shaped cells within the apical organ, is an ancestral condition in Gastropoda, and due to lifestyle demands variations might have evolved in the respective species (Fig. 12). Neritimorpha, in contrast, shows a clearly different morphology and pattern of cells within the apical organ, and thus might represent a secondary, derived condition that evolved within this particular clade (Fig. 12) (Page and Kempf 2009).

Besides serotonin other neuronal substances such as the FMRFamides or catecholamines have been identified within the apical organ in larvae of different gastropod clades (Dickinson et al. 1999, 2000; Voronezhskaya et al. 1999; Croll 2000; Pires et al. 2000; Dickinson and Croll 2003; Kristof and Klussmann‐Kolb 2010). Here, comparisons are difficult as the number of investigations utilizing antibodies against FMRFamides and tyrosine hydroxylase (enzyme involved in catecholamine biosynthesis) is considerably lower than against serotonin. Interestingly, some of the apical cells reported in the caenogastropods *Crepidula fornicata* and *Ilyanassa obsoleta* co‐express FMRFamides and serotonin (Dickinson et al. 1999; Dickinson and Croll 2003). In *L*. cf. *kogamogai*, however, the first FMRFamide‐containing neuronal structures appear, in relation to serotonin, notably later in development (trochophore vs. late veliger stage) in the *Anlagen* of the future cerebral ganglia and neither in the apical organ nor in the velum. In the heterobranchs *Lymnaea stagnalis*,* Aplysia californica* as well as in the ceanogastropods *C*. *fornicata* and *I*. *obsoleta*, posteriorly located FMRFamide‐lir cells appear as the first neuronal structures even before any immunoreactivity is noticeable within the apical organ or the velum (Croll and Voronezhskaya 1996; Dickinson et al. 1999, 2000; Croll 2000; Dickinson and Croll 2003). Moreover, it is assumed that the future adult central nervous system develops along the scaffold laid down by the neurites from those early, posterior FMRFamide‐lir cells (Croll and Dickinson 2004). It is questionable whether this is true for all gastropods, as this condition of early FMRFamide‐positive posterior cells scaffolding the adult central nervous system has not been reported for the nudipleuran *Aeolidiella stephanieae* (Kristof and Klussmann‐Kolb 2010) and the herein investigated patellogastropod *L*. cf. *kogamogai*. Additional information on development of FMRFamide‐lir elements in the nervous system of other gastropod taxa is clearly needed in order to gain more insights into the question whether or not the ‘scaffolding’ assumption might be true.

### Gastropod central nervous system development

*Lottia* cf. *kogamogai* shows notable differences in the appearance and distribution of neuronal elements containing serotonin and FMRFamides. For instance, serotonin is expressed in trochophore larvae early in neurogenesis (8 hpf), whereas FMRFamide‐positive neuronal elements appear considerably later in veliger or early pediveliger larvae (24–25 hpf). Noticeably, FMRFamide immunoreactivity appears later in development in the ganglia *Anlagen* of the future adult central nervous system, while earlier in development neural structures, such as the apical organ and the prototroch neurite ring, are immunoreactive to serotonin. As in numerous representatives from each of the five major gastropod clades (i.e. Patello‐, Veti‐, Caenogastropoda, Neritimorpha, Heterobranchia), immunoreactivity appears first in the apical organ in *L*. cf. *kogamogai* and then subsequently in the future adult cerebral and pedal ganglia as well as in the visceral ganglion (e.g. Barlow and Truman 1992; Kempf et al. 1997; Diefenbach et al. 1998; Page and Parries 2000; Page 2002a, 2006a; Page and Kempf 2009). On the contrary, in some caenogastropod and heterobranch species, the first FMRFamide‐lir cells appear in posterior ganglia well before the anterior cerebral and pedal ganglia show immunoreactivity, while, as mentioned above, immunoreactivity in ganglia follows an anterior to posterior sequence in the majority of gastropods (Croll and Voronezhskaya 1996; Dickinson et al. 1999, 2000; Dickinson and Croll 2003). However, only histological and ultrastructural studies might clarify whether gangliogenesis is directional or not, as neural structures immunoreactive to FMRFamide and serotonin do not represent the entire nervous system (Richter et al. 2010). For instance, *L*. cf. *kogamogai* shows no immunoreactivity in the more posterior ganglia such as the pleural, buccal or osphradial ganglia. These ganglia either appear later in development, possibly in juvenile stages, or are already present but lack the two investigated immunoreactive compounds, FMRFamide and serotonin.

In *L*. cf. *kogamogai* pediveliger larva (32 hpf), neurites immunoreactive against FMRFamides and serotonin appear first on the left side of the future visceral neurite loop, although the process of ontogenetic torsion has already occurred (indicated by the position of the main larval and accessory retractor muscles; Fig. 9). Only later in development (2 dpf) the crossing of the visceral neurites (streptoneury) is visible by antibodies against FMRFamide. This is not surprising as the different tissues and body parts are still in the process of differentiation after torsion has occurred (see Bondar and Page 2003; Page 2006a).

Interestingly, in the vetigastropod *Haliotis kamtschatkana* the pathway of the future visceral neurites is delineated before the onset of torsion by a posteriorly positioned serotonin‐lir cell (Page 2006a). This is neither the case in the closely related *Haliotis asinina* or *Haliotis rufescens* nor in the herein investigated patellogastropod *L*. cf. *kogamogai* (Barlow and Truman 1992; Hinman et al. 2003). As neurogenesis can differ even in closely related species, a multimethodological (e.g. ontogeny, immunohistochemistry, histology, TEM, SEM) as well as comparative approaches might be useful in order to enable reliable reconstructions of ancestral bodyplan features.

As in other gastropods, serotonin‐lir cells of *L*. cf. *kogamogai* are confined to the apical organ and the developing future adult ganglia and do not appear in peripheral areas such as the tentacles or the foot (e.g. Dickinson et al. 1999; Dickinson and Croll 2003; Wollesen et al. 2007; Kristof and Klussmann‐Kolb 2010). In contrast, FMRFamide‐containing cells appear not only in *Anlagen* of ganglia (cerebral, pedal, visceral ganglia) but also in the periphery (i.e. flask‐shaped cells at the base of the foot and the bipolar cells in the foot) (see Fig. 8). This is consistent with reports on numerous gastropods, where FMRFamide‐positive cells have been commonly described within the foot (Dickinson et al. 1999; Dickinson and Croll 2003; Wollesen et al. 2007; Kristof and Klussmann‐Kolb 2010). This is not surprising as the foot is a strong muscular structure and FMRFamides are assumed to be involved, among others, in the modulation of muscle activity and contraction (Cawthorpe and Lukowiak 1990; Evans et al. 1999). The FMRFamide‐lir flask‐shaped cells at the base of the foot have probably a different function than the FMRFamide‐lir cells within the foot. The sensory function of flask‐shaped cells is assumed due to the fact that they have a narrow opening to the external environment and a ciliated lumen (e.g. Chia and Koss 1984; Croll and Dickinson 2004; Page and Kempf 2009). Accordingly, the flask‐shaped cells at the base of the foot in the herein investigated *L*. cf. *kogamogai* might be involved in chemo‐ and or mechanosensory reception of the larva.

### Gastropod myogenesis

Larvae of numerous gastropods belonging to the five major clades (Patello‐, Veti‐, Caenogastropoda, Neritimorpha, Heterobranchia) have been investigated by F‐actin labelling in conjunction with laser scanning microscopy and/or by light and electron microscopy (e.g. Bonar and Hadfield 1974; Page 1997, 1998; Wanninger et al. 1999a; Ruthensteiner and Schaefer 2002; Evans et al. 2009; Page and Ferguson 2013).

In general, all pelagic gastropod larvae have one or two (main and accessory) larval retractor muscles (Fig. 13) that develop as first muscular structures in early larval stages and then later degenerate during or shortly after metamorphosis (Page 1997, 1998; Wanninger et al. 1999a; Wollesen et al. 2008; Kristof and Klussmann‐Kolb 2010; Page and Ferguson 2013). These larval retractor muscles are obliquely striated; while adult muscles, in contrast, are usually smooth (Page 1995, 1997, 1998; Evans et al. 2009; Page and Ferguson 2013). In *Lottia* cf. *kogamogai* both larval retractor muscles anchor ventro‐posteriorly to the inner wall of the protoconch and project into the prototroch, the anterior portion of the forming foot (propodium) and the mantle, as reported for the larval retractor muscles in other patellogastropods (Wanninger et al. 1999a; Damen and Dictus 2002), vetigastropods (Degnan et al. 1997; Page 1997, 2002b), the ceanogastropod *Ilyanassa obsoleta* (Evans et al. 2009), and some euthyneurans (Horikoshi 1967; Smith 1967; Bonar and Hadfield 1974; Wollesen et al. 2008; Kristof and Klussmann‐Kolb 2010). The main larval retractor muscle in post‐torsional larvae generally anchors posteriorly and slightly left of the midline at the inner wall of the protoconch. From that attachment site, muscle fibres or bands of the retractor extend mainly into the prototroch or velar lobes and in parts also into the foot. In contrast, the accessory larval retractor muscle, if present, mainly projects into the mantle and in some cases also into the prototroch/velum. In addition, the attachment site at the protoconch varies in different gastropod larvae (Fig. 13). The accessory larval retractor muscle inserts in a ventro‐anterior position with respect to the main larval retractor in patello‐ and vetigastropods as well as in the nudipleuran *Phestilla sibogae* and in a dorsal position in the nudipleuran *Aeolidiella stephanieae* and the ceanogastropod *I. obsoleta* (Bonar and Hadfield 1974; Page 1997, 2002b; Wanninger et al. 1999a; Damen and Dictus 2002; Evans et al. 2009; Kristof and Klussmann‐Kolb 2010). Interestingly, the neritimorph gastropod *Nerita melanotragus* exhibits two larval retractor muscles of which the left muscle projects into both velar lobes and is distinctly larger than the right muscle (Page and Ferguson 2013). In addition, the larval retractor muscles share morphological similarities (striation pattern, insertion sites, projection into velar lobes and the base of the foot) with those in other gastropod larvae. Therefore, it seems likely that the right and left retractor muscles are homologous to the accessory and main larval retractor muscle in the above‐mentioned gastropods, respectively. Although three larval retractor muscles are described in larvae of *Fiona marina* (Nudipleura) only one of them inserts at the posterior end of the protoconch (left to the midline) and projects into the velum (Casteel 1904). The additional two ‘larval retractor muscles’ are in fact pedal retractors, as they insert at the protoconch on each lateral side posterior to the base of the foot and project into the same (Casteel 1904). Accordingly, *F*. *marina* larvae exhibit one main larval retractor muscle, as it is also the case in all panpulmonate and numerous other euthyneuran and caenogastropod larvae (Fig. 13) (Casteel 1904; Werner 1955; Thompson 1958; Kawaguti and Yamasu 1960a,b; D'Asaro 1965, 1966; Fretter 1972; Bridges 1975; Page 1995, 1998, 2006b; Ruthensteiner and Schaefer 2002). As Euthyneura and Caenogastropoda are considered as derived gastropod taxa (Haszprunar 1988; Ponder and Lindberg 1997; McArthur and Harasewych 2003; Aktipis et al. 2008; Smith et al. 2011; Kocot et al. 2011; Osca et al. 2014; Schrödl 2014; Zapata et al. 2014), it seems likely that the condition with two obliquely striated, shell attached larval retractor muscles as found in the basal branching clades such as Patellogastropoda and Vetigastropoda represents the ancestral condition for Gastropoda.

In contrast to the larval retractor(s), the pedal retractor muscle is smooth and forms later in development. It contributes to the postmetamorphic adult musculature, where it forms (the *Anlage* of) the adult shell or collumellar muscle(s) (e.g. Page 1997, 1998; Wanninger et al. 1999a,b; Wollesen et al. 2008; Evans et al. 2009). This is congruent with our observations in *L*. cf. *kogamogai* where both larval retractor muscles as well as the velum muscle ring degenerate towards metamorphosis, while the pedal retractor muscle increases massively in size. This coincides with a behavioural transition of the larvae from swimming to crawling, which is followed by settlement and metamorphosis. As in other investigated patellogastropds, some juvenile muscles such as the tentacle and foot musculature develop prior to metamorphosis, while others such as the buccal musculature form afterwards (Wanninger et al. 1999a; Damen and Dictus 2002). In the caenogastropod *I*. *obsoleta*, the buccal and siphon musculature develops prior to metamorphosis, while in euopisthobranchs and nudipleurans the buccal musculature consistently develops well after metamorphosis (Bonar and Hadfield 1974; Wollesen et al. 2008; Evans et al. 2009; Kristof and Klussmann‐Kolb 2010). These differences in the appearance of juvenile musculature reflect the life history or ecology of the respective species.

Interestingly, *L*. cf. *kogamogai* larvae show a pair of muscle bundles (see Fig. 10; cephalopedal muscles) that inserts at the base of the metapodium and projects through the cephalic region towards the tentacles. Such a muscle system has not been described in any other gastropod larva to date. A pair of transverse muscle fibres was reported for the first time in *Patella coerulea* larvae (Damen and Dictus 2002). These muscle fibres form a U with the base of the U at the ventral side and are connected to the lateral shell walls and are assumed to act antagonistically to the larval retractor muscles (Damen and Dictus 2002). These muscle fibres in *P*. *coerulea* do not correspond to the cephalopedal muscles described herein in *L*. cf. *kogamogai* as they have a different insertion (medio‐ventral location between the foot and the visceral mass *versus* base of the metapodium) and projection site (dorsal part of the visceropallium *versus* dorsal part of the cephalopodium). However, it is not clear whether these cephalopedal muscles in *L*. cf. *kogamogai* or the transverse muscles in *P*. *coerulea* contribute to the adult muscle complex or not. Damen and Dictus (2002) assumed that the accessory retractor muscle, which projects dorsally into the mantle, is the main extensor that together with the transverse muscles forms the antagonists of the main larval retractor muscle. The contraction of the main larval retractor retracts the larval body into the shell just as the extensors at contraction expand it from the shell (*sensu* Damen and Dictus 2002). Regardless of their function, both, the main and accessory retractor muscles degenerate during metamorphosis. As the transverse muscles in *P*. *coerulea* might also belong to the larval muscle system it appears plausible that they disintegrate during metamorphosis as well. However, as the cephalopedal muscles in *L*. cf. *kogamogai* are not homologous to the transverse muscles in *P*. *coerulea* their function and fate remains highly speculative. Whether these cephalopedal muscles in *L*. cf. *kogamogai* contribute to the adult musculature (possibly as cephalic retractors) or are truly larval and disappear during metamorphosis could not be determined. Future investigations will have to provide data whether or not the transverse muscles such as in *P*. *coerulea* or the cephalopedal muscles as in *L*. cf. *kogamogai* are also common in other gastropods.

### Neuromuscular patterns in Gastropoda and other molluscs

Besides Gastropoda, an apical organ with serotonin and FMRFamide‐positive immunoreactive cells has also been reported for numerous other molluscan taxa such as Bivalvia, Scaphopoda, Polyplacophora, and Solenogastres (Kreiling et al. 2001; Friedrich et al. 2002; Voronezhskaya et al. 2002, 2008; Wanninger and Haszprunar 2003; Dyachuk and Odintsova 2009; Redl et al. 2014). The largest number of immunoreactive cells within the apical organ among all molluscs is reported for polyplacophoran larvae, where eight serotonin‐lir and up to six FMRFamide‐lir flask‐shaped cells are present (Friedrich et al. 2002; Voronezhskaya et al. 2002). Within Bivalvia, the number of serotonin‐ and/or FMRFamide‐positive cells varies from five (*Mytilus trossulus*) to three (*Spisula solidissima*; only serotonin) flask‐shaped apical cells, while the scaphopod *Antalis entalis* exhibits four serotonin‐lir flask‐shaped and no FMRFamide‐lir cells within the apical organ (Kreiling et al. 2001; Wanninger and Haszprunar 2003; Voronezhskaya et al. 2008; Dyachuk and Odintsova 2009). Recently, the solenogaster *Wirenia argentea* was shown to possess eight apical flask‐shaped cells (stained by anti‐alpha‐tubulin) of which two are serotonin‐lir and additional two FMRFamide‐lir (Redl et al. 2014). Similar condition of apical organs with up to four flask‐shaped cells have also been reported in most other lophotrochozoans such as annelids, nemerteans, ectoprocts and the brachiopod *Novocrania anomala* (Voronezhskaya et al. 2003; Kristof et al. 2008; Gruhl 2009; Altenburger and Wanninger 2010; Chernyshev and Magarlamov 2010; Hindinger et al. 2013). On the other hand, conditions similar to that found in Polyplacophora where numerous serotonin‐lir flask‐shaped cells are present within the apical organ have also been reported for the creeping‐type larva of entoprocts (Kamptozoa) (Wanninger et al. 2007; Wanninger 2009), the brachiopod *Terebratalia transversa* (Altenburger et al. 2011), and the actinotroch larva of phoronids (Santagata 2002; Temereva and Wanninger 2012; Sonnleitner et al. 2014). This indicates that most probably in the last common lophotrochozoan ancestor the apical organ was simple and comprised up to four flask‐shaped, serotonin and/or FMRFamide expressing cells (Wanninger 2009). Regardless the number of cells within the apical organ, the cerebral commissure generally develops at its base, suggesting an inductive role in the formation of the future adult nervous system.

*Lottia* cf. *kogamogai* larvae exhibit a serotonin‐lir neurite ring that underlies the prototroch. Such a neurite ring that underlies ciliated bands appears to be common in gastropods, solenogasters, polyplacophorans as well as in various other lophotrochozoans (Gastropoda: Kempf et al. 1997; Dickinson et al. 1999; Page and Parries 2000; Page 2002a, 2006a; Dickinson and Croll 2003; Wollesen et al. 2007; Kristof and Klussmann‐Kolb 2010; Solenogastres: Redl et al. 2014; Polyplacophora: Friedrich et al. 2002; Voronezhskaya et al. 2002; Annelida: Brinkmann and Wanninger 2008, 2009; Kristof et al. 2008; Helm et al. 2013; Nemertea: Hindinger et al. 2013; Bryozoa: Wanninger et al. 2005; Gruhl 2009; Entoprocta: Wanninger et al. 2007; Nielsen and Worsaae 2010; Phoronida: Santagata 2002; Temereva and Wanninger 2012; Sonnleitner et al. 2014; Temereva and Tsitrin 2014). The lack of that feature in ecdysozoans and non‐bilaterians suggest that this is an apomorphic condition for Lophotrochozoa (see Wanninger 2009, 2015a,b for reviews). Hence, the fact that the serotonin‐lir (or FMRFamide‐lir) prototrochal neurite has not been reported in the investigated scaphopod *Antalis entalis* and bivalves *Spisula solidissima* and *Mytilus trossulus* suggests a secondary loss in these molluscs (Kreiling et al. 2001; Wanninger and Haszprunar 2003; Voronezhskaya et al. 2008).

Bivalve and gastropod larvae show striking similarities in their larval musculature such as the velum retractor muscles and the prototroch or velum muscle ring. In general, the larval retractor muscles are striated, insert posteriorly at the inner shell, project into the velum, foot or mantle, and in almost all cases degenerate during metamorphosis (Degnan et al. 1997; Page 1998; Wanninger et al. 1999a; Wollesen et al. 2008; Dyachuk and Odintsova 2009; Kristof and Klussmann‐Kolb 2010; Page and Ferguson 2013; Wurzinger‐Mayer et al. 2014). The muscle ring that underlies the prototroch or velum is not only found in the patellogastropod *L*. cf. *kogamogai* but also in other gastropods, bivalves, polyplacophorans, solenogastres, and caudofoveates as well as in annelids and entoprocts (e.g. Page 2002b; Wanninger and Haszprunar 2002a; Nielsen et al. 2007; Wollesen et al. 2008; Dyachuk and Odintsova 2009; Evans et al. 2009; Wanninger 2009; Page and Ferguson 2013; Scherholz et al. 2013; Wurzinger‐Mayer et al. 2014; Merkel et al. 2015). The absence of such a prototrochal muscle ring in Scaphopoda suggests, once again, a secondary loss (Wanninger and Haszprunar 2002b).

## Conclusions

A similar morphology and arrangement of neural and muscular elements is found in larvae throughout the five major gastropod clades. As shown in this study, the patellogastropod *Lottia* cf. *kogamogai* shares a number of bodyplan features that are conserved among gastropods, namely the five serotonin‐lir apical cells, the prototrochal neurite and muscular ring, and two larval retractor muscles. Therefore, these characters can be considered as part of the body plan of the larva of the last common gastropod ancestor. Although some of these characters are also found in various lophotrochozoans such as serotonin‐lir and/or FMRFamide‐lir apical flask‐shaped cells and the prototrochal neurite and muscular ring, the specific arrangement of five (two round, one median and two lateral flask‐shaped) serotonin‐lir apical cells has so far only been found in gastropod larvae. The evolutionary changes in, for instance, the mode of development, have probably led to adaptations and/or secondary losses of some neural and muscular elements in various gastropod lineages. However, gastropods and bivalves are the only molluscs that exhibit prototroch or velar retractor muscles, while these are absent in scaphopods, cephalopods, polyplacophorans, solenogastres and caudofoveates. This might be correlated with the presence of an embryonic shell in Gastropoda and Bivalvia. Although an embryonic shell is formed in Scaphopoda, such larval retractor muscles are missing. Whether these larval retractor muscles have evolved independently in Gastropod and Bivalvia or are homologous and have been lost in Scaphopoda remains to be shown.
